# The Morphologies of the Semiconductor Oxides and Their Gas-Sensing Properties

**DOI:** 10.3390/s17122779

**Published:** 2017-11-30

**Authors:** Tingting Lin, Xin Lv, Shuang Li, Qingji Wang

**Affiliations:** 1 College of Instrumentation and Electrical Engineering, Jilin University, Changchun 130061, China; ttlin@jlu.edu.cn (T.L.); xinlv15@mails.jlu.edu.cn (X.L.); shuangli16@mails.jlu.edu.cn (S.L.); 2 Key Laboratory of Geophysics Exploration Equipment, Ministry of Education of China, Changchun 130061, China

**Keywords:** morphology, nanostructure, semiconductor, oxide, gas sensor

## Abstract

Semiconductor oxide chemoresistive gas sensors are widely used for detecting deleterious gases due to low cost, simple preparation, rapid response and high sensitivity. The performance of gas sensor is greatly affected by the morphology of the semiconductor oxide. There are many semiconductor oxide morphologies, including zero-dimensional, one-dimensional, two-dimensional and three-dimensional ones. The semiconductor oxides with different morphologies significantly enhance the gas-sensing performance. Among the various morphologies, hollow nanostructures and core-shell nanostructures are always the focus of research in the field of gas sensors due to their distinctive structural characteristics and superior performance. Herein the morphologies of semiconductor oxides and their gas-sensing properties are reviewed. This review also proposes a potential strategy for the enhancement of gas-sensing performance in the future.

## 1. Introduction

In recent years, the issue of environmental safety has attracted much attention with rapid economic development [1,2,3,4]. The activities in our everyday life result in the release of many deleterious gases, such as H_2_S [5], NO_2_ [6], CO [7], HCHO [8], H_2_ [9], NH_3_ [10], CO_2_ [11], C_2_H_2_ [12], C_2_H_5_OH [13], etc. It is not merely that these gases harm people’s health, but they also restrict sustainable development in the current situation. In order to effectively detect various gases, many researchers have tried to develop different kinds of gas sensors. Among the numerous gas sensors, semiconductor oxide gas sensors have received great attention owing to their high selectivity and sensitivity, and fast response for monitoring hazardous gases.

As we all know, the sensing material is the core component of semiconductor oxide gas sensors, and the morphologies of semiconductor oxides have a critical influence on the gas-sensing performance of sensors. The gas-sensing performance is determined by the reaction rate and amount of oxygen species in the redox reaction, which depend on the microstructure, shape and size of the semiconductor oxide used. In addition, the sensing process occurs on the different sites of the sensing material and thus the performance is related to the morphology. Furthermore, different morphologies have different advantages, contributing to increased surface active sites, accelerated response speeds and enhanced gas diffusion.

Many researchers have attempted to fabricate novel nanostructures to improve the performance of sensors [14,15]. Various semiconductor oxide morphologies have been prepared, including nanoparticles [16], nanowires [17], nanofibers [18], nanosheets [19], nanoflowers [20], hollow [21] and core-shell [22] nanostructures, etc. These semiconductor oxides are commonly prepared by two methods: top-down techniques and the bottom-up techniques. In the top-down techniques, the specific size and morphology of nanomaterials can be obtained from larger or bulk solids. By contrast, the bottom-up techniques construct nanostructures through crystal growth and self-assembled units. So far, many reviews about semiconductor oxide gas sensors have been published [23,24,25,26]. Nevertheless, the relation between the morphology of semiconducting oxide and the gas-sensing performance of the sensor using them are not clearly established yet. Therefore, the understanding of favorable morphologies and the parameters of the microstructure at the interaction with gases are important for further improvement of gas sensors. In this review, we provide an overview of semiconductor oxides with different morphologies with gas-sensing properties, with the expectation of providing potential guidelines for identifying morphology characteristics that at beneficial for sensing performance.

## 2. Gas-Sensing Mechanism

To better design novel semiconductor oxide morphologies and improve the gas-sensing performances of sensors, it is necessary to clarify the gas-sensing mechanism. As we all know, the working principle of gas sensors is based on the change in resistance of the sensor on exposure to a target gas. However, the gas-sensing mechanism details are still disputed. This review will present five aspects to understand the gas-sensing mechanism, including the adsorption of oxygen on the surface of semiconductor oxides, band gap, Schottky barrier contact, the catalysis-based sensing mechanism and the heterojunction-based sensing mechanism.

### 2.1. Adsorption Oxygen on the Surface of Semiconductor Oxides

The adsorption reactions occur on the surface of semiconductor oxides, and depend on the type of target gas. In an air atmosphere, oxygen adsorption on the oxide surface forms different absorbed oxygen species (O_2_^−^, O^−^ or O^2−^). The different oxygen species depend on the working temperature [27]. The process is described as the following equations:

(1) $$O_{2}(\mathrm{gas})\to O_{2}(\mathrm{absorbed})$$

(2) $$O_{2}(\mathrm{absorbed})+e^{-}\to O_{2}{}^{-}$$

(3) $$O_{2}{}^{-}+e^{-}\to 2O^{-}$$

(4) $$O^{-}+e^{-}\to O^{2-}$$

**Figure 1:**
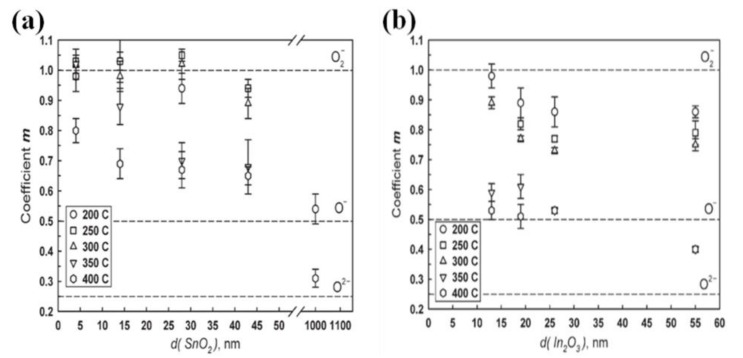
Calculated values of coefficient *m*. (**a**) SnO_2_ samples, (**b**) In_2_O_3_ samples. Reprint from [28] with permission. Copyright (2009) Elsevier.

Apart from the working temperature, different oxygen species are also determined by the grain size. Rumyantseva et al. have investigated the correlations between grain size and oxygen chemisorption for nanocrystalline SnO_2_ and In_2_O_3_ [28]. The values of coefficient *m* changes with grain size at 200–400 °C, as shown in Figure 1a,b. The coefficient *m* value decreases with increasing grain size. When *m* = 1, oxygen chemisorption mainly exists in the form of molecular (O^2−^). When *m* = 0.5, oxygen species mainly take the form of atomic O^−^. When *m* = 0.25, atomic O_2_^−^ dominates in the oxygen chemisorption. It can be seen that decrease of *m* increases the amount of the atomic forms, so grain size enlargement increases the fraction of atomic forms of absorbed oxygen species. Obviously, oxygen chemisorption is affected by the grain size.

The semiconducting oxides can be divided into n-type and p-type depending on the type of majority carrier. Most semiconductor oxide gas sensors are n-type, in which the majority carriers are electrons. For reducing gases in the adsorption process of semiconductor oxides, reductive gases will react with the adsorbed oxygen species and decrease the resistance of the sensor, but when the sensor is exposed to oxidizing gases at a feasible temperature, the gas molecules can take away electrons to form ion gases and react with the chemisorbed oxygen on the surface, leading to changes in the resistance of the sensor.

### 2.2. Band Gap

**Figure 2:**
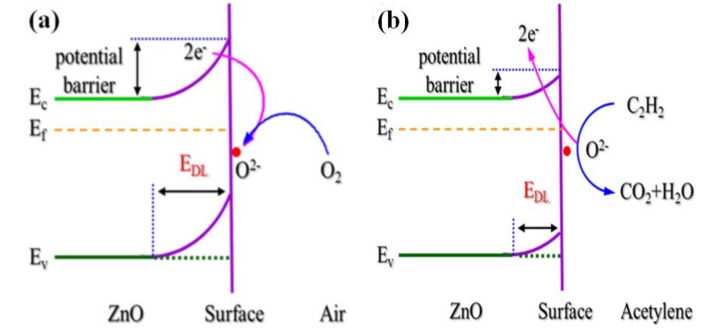

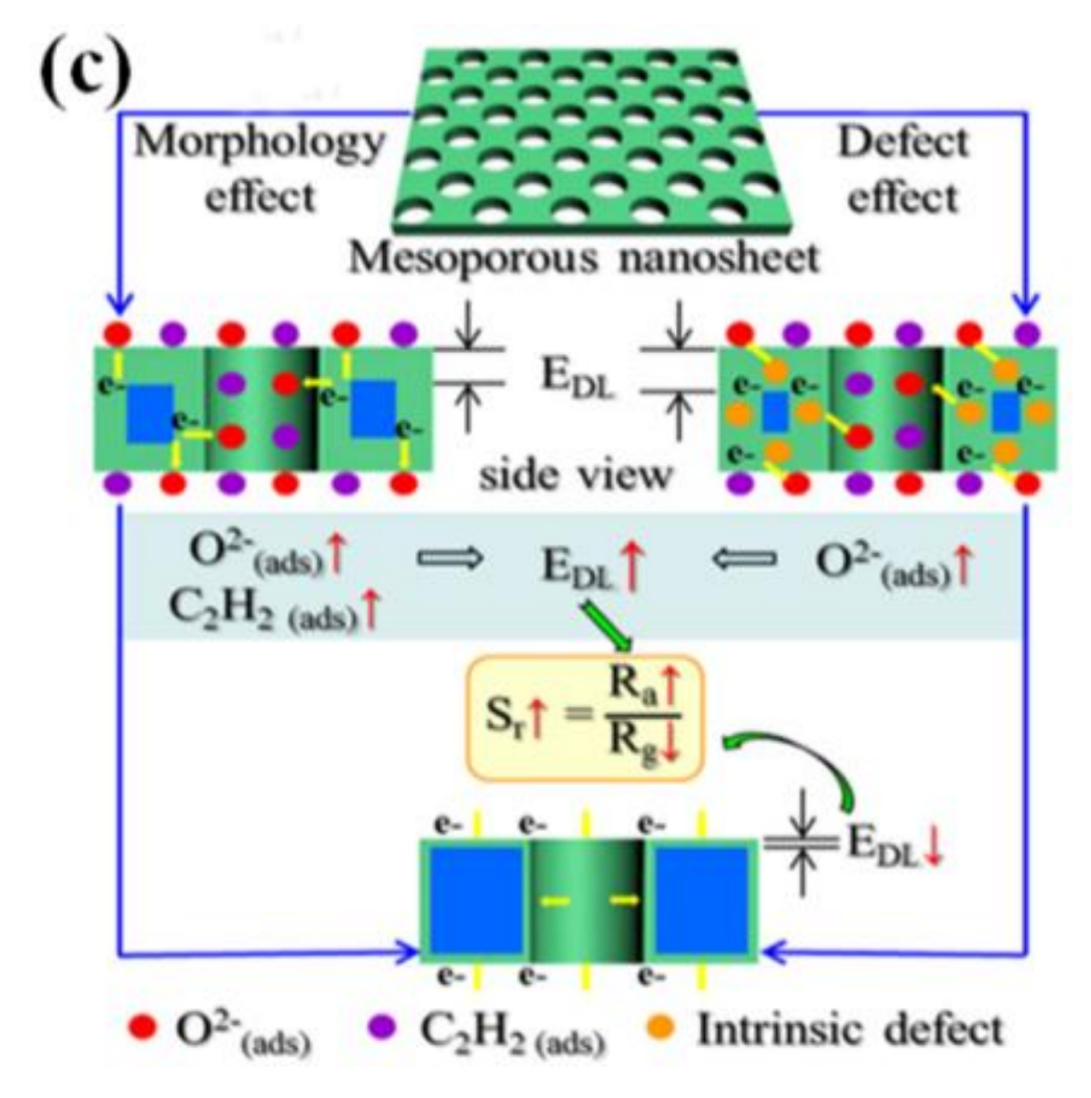
Schematic illustration of acetylene sensing mechanism for ZnO sensor based on the band model. (**a**) in air; (**b**) in acetylene; (**c**) morphology and defect effects. Reprinted from [29] with permission. Copyright (2017) Elsevier.

The electrical properties are affected by the band gap of semiconductor oxides, so the band gap theory is also adopted on the sensing mechanism of gas sensors. Qiao et al. investigated the gas-sensing mechanism of mesoporous ZnO nanosheets based on the band model [29]. In air, oxygen molecules would absorb on the surface of ZnO nanosheets to form oxygen ionic species by trapping electrons from the conduction band, synchronously creating an electron depletion zone and upward band bending, as shown in Figure 2a. This variation of band bending also alters the resistance of the sensor. When exposed to an acetylene gas atmosphere, adsorbed oxygen ions would react with the reducing gas and release the trapped electrons back to the conduction band, leading to a reduced thickness of the electron depletion layer and decreasing the resistance of the sensor, as shown in Figure 2b. Moreover, Qiao et al. proposed that the microstructure affects the acetylene sensing mechanism, that is to say, the synergetic effects of morphology and defect enhanced sensing mechanism of a mesoporous ZnO nanosheet, as shown in Figure 2c. On the one hand, the adsorption capacity for gas molecules was increased owing to the large specific surface area and gas diffusion was enhanced due to the mesoporous nanostructure. Besides, the thickness of the ZnO nanosheets was nearly identical to two times the Debye length, resulting in a fully depleted shell layer and an enhanced gas-sensing performance. On the other hand, intrinsic defects in the ZnO nanosheet act as active sites for oxygen chemisorption and ionization of oxygen species on the surface of ZnO nanosheet-based sensor, making the sensing reaction go forward in the positive direction, so abundant intrinsic defects lead to more ionized oxygen species. When acetylene gas was injected, more electrons go back to the conduction band, resulting in a lower resistance. Therefore, the enhanced sensing mechanism was attributed to the morphology and defects.

### 2.3. Schottky Barrier Contact

**Figure 3:**
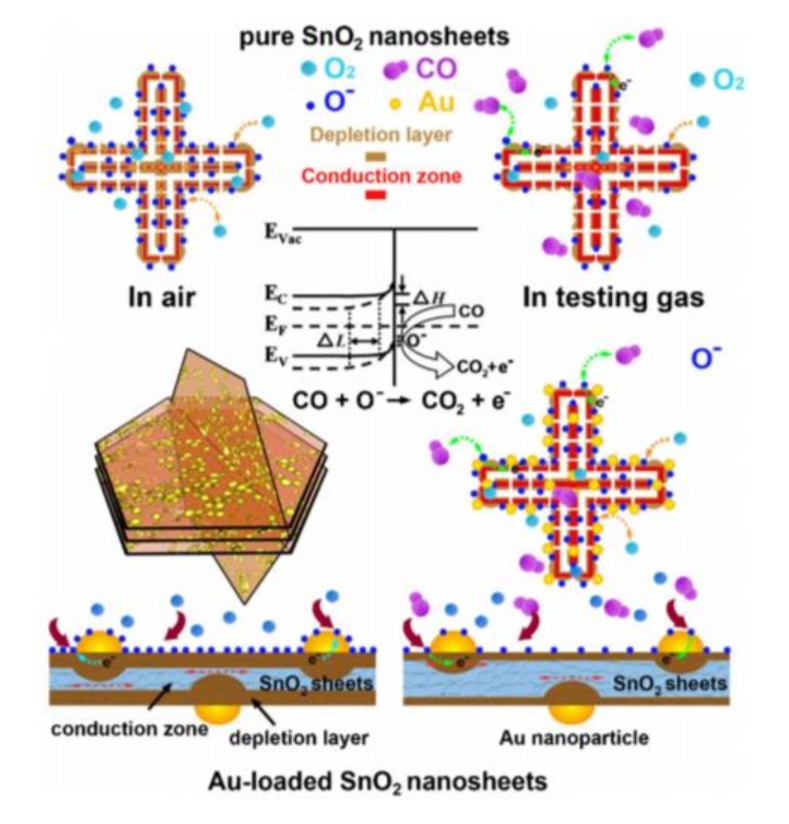
Schematic view of the sensing mechanism and surface processes associated with the reaction with ambient oxygen (**left**) and testing CO (**right**). Reprinted from [31] with permission. Copyright (2015) Elsevier.

Schottky barrier is understood to be a contact between a metal and a semiconductor oxide coming from two different work functions [30]. The combination of noble metal and semiconductor oxide can help improve the gas-sensing performance. Yao et al. investigated the signal enhancement mechanism of Au/SnO_2_ hollow multilayered nanosheets [31]. The work function of Au was larger than that of SnO_2_, so the electrons are transferred from SnO_2_ to Au until equilibrium is achieved. In air, a Au/SnO_2_ hollow multilayered nanosheet would interact with oxidizing gas, and more electrons are trapped from the Au/SnO_2_ hollow multilayered nanosheet surface. In this process, the depletion layer of Au/SnO_2_ became wider (ΔL) and higher (ΔH) than that of pristine SnO_2_ due to the Au/SnO_2_ junction coming from Schottky barrier contacts, and the resistance of the sensor increases correspondingly, as shown in Figure 3. For the reaction between Au/SnO_2_ hollow multilayered nanosheets and CO, the resistance of the sensor becomes lower due to the accumulation of more electrons induced by the nano-Schottky junction between Au and SnO_2_.

In addition, the improvement of gas-sensing performance could be attributed to the morphology. The hollow nanostructure and multilayered wall could offer higher surface-to-volume ratios and facilitate gas diffusion, leading to high response and faster response times. Moreover, the unique structure could absorb gases into the inner-surface/outer-surface, resulting in improved gas-sensing performance.

### 2.4. Catalysis-Based Sensing Mechanism

The semiconductor oxides with an added noble metal can accelerate the reaction process and improve the gas sensor performance. The catalyst can enhance the performance of a sensor in two ways, namely chemical sensitization and electronic sensitization [32]. In the chemical sensitization aspect, the noble metal catalyst facilitates the redox reaction occurring on the surface due to the spillover effect. In this process, more oxygen molecules will absorb on the surface of the semiconductor oxide and drain more free electrons from the semiconductor oxide surface to form adsorbed oxygen species. In the electronic sensitization aspect, the noble metal catalyst acts as an electron acceptor on the surface of the semiconductor oxide, and more electrons will be transferred from the conduction band of the semiconductor oxide, resulting in a greater energy band bending and a broader depletion layer. Thus, the variation of resistance in the catalysis-based semiconductor oxide is larger than that of bare semiconductor oxide, which leads to the increase of the gas-sensing performance.

**Figure 4:**
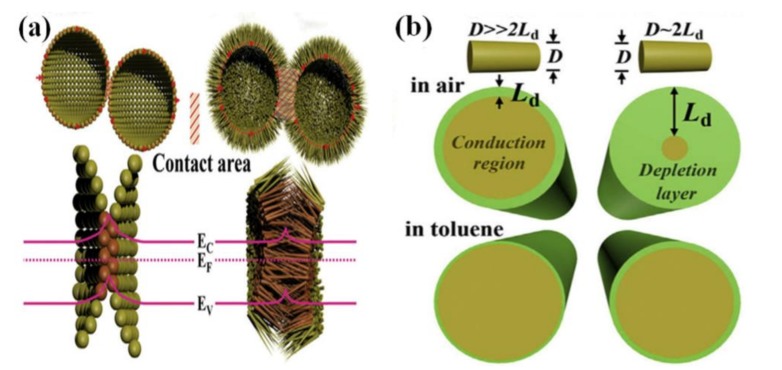
Schematic of catalysis-based sensing mechanism in the nanorod-assembled porous SnO2 hollow microspheres. (**a**) interface region associated with the point-to-point (**left**) and multipoint contacts (**right**); (**b**) schematic view of depletion layer on the cross section from nanorods with different diameters. Reprinted from [33] with permission. Copyright (2016) Elsevier.

In addition, the morphology and parameters of the microstructure affect the catalysis sensing process. Zhang et al. indicated that nanorod-assembled porous SnO_2_ hollow microspheres offered numerous channels for gas diffusion and absorbed more gases on the inner and outer surface [33], so Pd could promote more oxygen dissociation in the chemical sensitization effect. The porous structures accelerate gas diffusion and shorten the response time. Compared to traditional spheres with point-to-point contacts, nanorod-assembled microspheres with multipoint contacts increased the contact area, as shown in Figure 4a. Therefore, there was a wider conductive path for more electrons to transfer in the electronic sensitization effect. The sensing reaction is influenced by the relation between nanorod diameter D and the Debye length L_d_. When the diameter D is nearly identical to the Debye length L_d_, the free electrons were almost fully depleted resulting in changes of the whole conductivity. The ultrathin nanorod had a small diameter of about 10 nm, and the calculated Debye length was about 6.6 nm, so the ultrathin nanorod meets the condition of D ~ 2L_d_, and the amount of ionized oxygen would be maximized, as shown in Figure 4b.

### 2.5. Heterojunction-Based Sensing Mechanism

**Figure 5:**
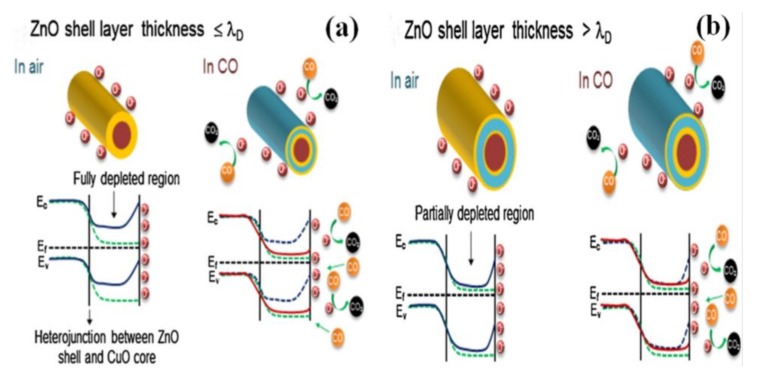
Schematic of heterojunction-based sensing mechanism in the CuO–ZnO core–shell nanowires. (**a**) thinner than ZnO’s Debye length; (**b**) thicker than ZnO’s Debye length. Reprinted from [35] with permission. Copyright (2015) Elsevier.

Semiconductor oxide heterostructures have been synthesized for the improvement of gas-sensing performances, such as p-p, n-n and p-n junctions. The heterostructure consists of two semiconductor oxides and they have different Fermi energy levels. When they contact with each other, free electrons transfer from the higher Fermi energy level to the lower Fermi energy level. The results indicate that a drain of free electrons occurs above the two semiconductor oxides, creating thicker depletion layers and higher resistance at the interface compared with that of a single oxide [34]. As a result, the introduction of a heterojunction has been carried out to improve the gas-sensing performance of sensors. Kim et al. investigated the role of the shell thickness of CuO/ZnO core-shell nanowires in the sensing mechanism, as shown in Figure 5. In the case where the shell thickness was nearly identical to λ_D_ of the shell material, a complete electron depletion was generated [35]. When exposed to CO gas, the adsorbed oxygen species would react with CO gas and release the trapped electrons back into the conduction band to recover its original configuration, leading to an enhanced gas response, as shown in Figure 5a. In another case, the shell thicker than λ_D_ would have only partial electron depletion. When the sensor is exposed to CO gas, CO gas will absorb on the partially depleted shell layer. The resistance of the sensor has a small change, as shown in Figure 5b, so controlling the shell thickness could achieve superior gas-sensing performance.

## 3. Different Morphologies of Nanomaterials for Gas-Sensing Performances

Many semiconductor oxides have been widely used in gas sensors, such as SnO_2_ [36], ZnO [37], WO_3_ [38], In_2_O_3_ [39], NiO [40], TiO_2_ [41], etc. In recent years, enormous efforts have been made to fabricate various semiconductor oxide morphologies to improve the gas-sensing performances. The morphologies of semiconductor oxides can be classified by dimensionality in some papers [42,43,44]. On the basis of this classification, semiconductor oxides can be defined as zero-dimensional, one-dimensional, two-dimensional and three-dimensional nanostructured materials. We know that semiconductor oxides with different morphologies have different natures, so we need to get some insight to know the relation between the morphologies and their gas-sensing properties.

### 3.1. Zero-Dimensional Sensing Materials

Zero-dimensional nanoparticles possess a special surface area to adsorb a target gas, which provides an opportunity to improve the gas-sensing performance [45,46,47,48,49]. In addition, the sensitive surface, agglomeration and size of nanoparticle can be adjusted to improve the sensing performance. The gas-sensing reactions occur on the active sites of the surface through gas adsorption, charge transfer and desorption. Consequently, the amount of surface active sites has the possibility to influence the gas response, which is ascribed to improved oxygen adsorption arising from the increment of surface active sites. The increment of surface active sites can be achieved by increasing the specific surface area. This prompts more target gas molecules to participate in the oxidation and reduction reaction. Here we discuss Cu_2_O nanoparticles and their gas-sensing performance. Wang et al. prepared concave Cu_2_O octahedral nanoparticles with 400 nm in diameter, which displayed better gas-sensing performance in the detection of benzene and nitrogen dioxide than Cu_2_O nanorods [50]. As a p-type semiconductor, Cu_2_O nanoparticles react with oxidizing gases (NO_2_), which exhibit a low resistance value, but Cu_2_O nanoparticles interact with a reducing gas (C_6_H_6_) representing an opposite trend in resistance. Unlike traditional octahedral structures, Cu_2_O octahedral nanoparticles were akin to icositetrahedron structures with distinctly boundaries, so Cu_2_O nanoparticles with eight sunken faces had a greater specific surface area than the nanorod one. A higher specific surface area provides more active sites for the surface reaction, which was responsible for a higher and faster gas response. Recently, colloidal quantum dots (CQDs) are used in gas sensors due to their large sensitive surface with precise control over nanoparticle size. Based on the advantages of CQDs, the WO_3_ CQDs-based sensor was exploited to detect H_2_S gas, for which it possessed a fast response and reduced power consumption at an optimal temperature as low as 80 °C, owing to the extremely small crystal size allowing a stronger surface effect [51].

**Figure 6:**
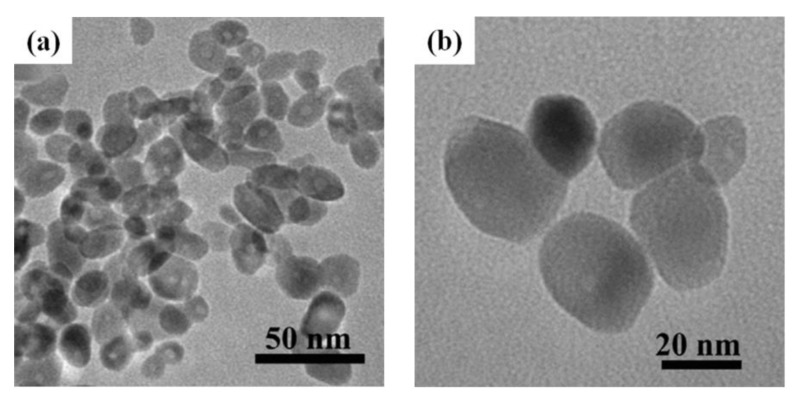

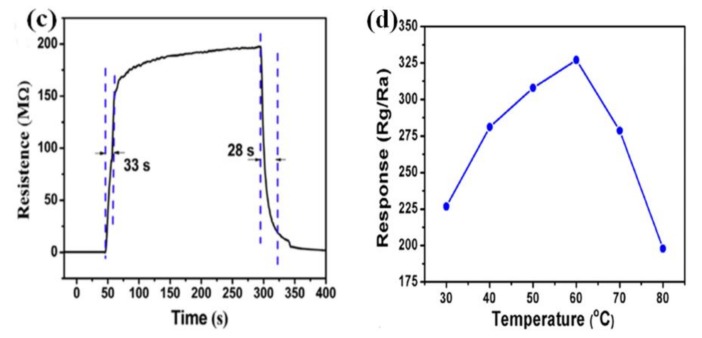
(**a**,**b**) TEM images of the In_2_O_3_ nanoparticles; (**c**) the response and recovery times of In_2_O_3_ nanoparticle sensor towards 20 ppm NO_2_; (**d**) Response of the sensor based on In_2_O_3_ nanoparticles to 5 ppm NO_2_. Reprinted from [52] with permission. Copyright (2016) Elsevier.

In general, conglomeration of nanoparticles has been deemed as a factor that has an effect on the sensing performance, restricting the applications of gas sensors. Because there is no guarantee that semiconductor oxides are adequately responsive to target gases in the presence of agglomeration, therefore, the uniform distribution of nanoparticles is important for gas-sensing reactions. Xiao et al. have prepared In_2_O_3_ nanoparticles, having a uniform size with unique diameter (20–30 nm), as shown in Figure 6a,b . It could be clearly seen that the nanoparticles are dispersed evenly. The gas sensor based on In_2_O_3_ nanoparticles exhibits fast and high response at a rather low operating temperature (60 °C), as shown in Figure 6c,d [52]. This may be attributed to the surface morphology of the nanoparticles. The uniformly dispersed nanoparticles with relatively small size could ensure quick responsiveness. Thus, the In_2_O_3_ nanoparticles as gas sensors have potential for the detection of NO_2_. In addition, researchers have always endeavored to obtain tiny enough nanoparticles on account of the small size effect. Based on Xu’s model [53], the response of the sensor increases remarkably if the particle size (D) of semiconductor oxide is less than or equal to double the width of the space charge layer (L). To investigate the small size effect, Liang et al. have prepared highly monodisperse α-Fe_2_O_3_ nanoparticles about 3 nm in size by a reverse micro-emulsion method [54]. On the surface of α-Fe_2_O_3_ nanoparticles, oxygen molecules drained electrons from the conduction band and become ionized to O_2_^−^, O^−^ or O^2−^, which created an electron depleted region called the space charge layer. The width of the space charge layer is about 44 nm. It was evident that the α-Fe_2_O_3_ nanoparticles met the condition of D ≤ 2L, so the whole grains were almost fully depleted resulting in changes to the whole conductivity, thus having the capability to enhance the acetone sensing performance, and showing that zero-dimensional nanoparticles exhibit superior sensing abilities for target gases.

### 3.2. One-Dimensional Sensing Materials

One-dimensional nanostructured materials with high aspect (length divided by width) ratio are potential candidates for gas sensors. The lengths of 1D semiconductor oxide particles can be a few millimeters long, but the thickness and widths are only within a limited range between 1 and 100 nm [55]. More and more people are very enthusiastic about fabricating 1D nanostructures in the forms of nanowires, nanotubes, nanobelts, nanorods, nanofibers, nanobundles, etc. [56,57,58,59,60,61,62,63,64,65,66,67]. These 1D nanomaterials with enhanced gas-sensing properties are suitable for gas sensors. The gas-sensing performances of 1D nanostructures with various morphologies are summarized in Table 1.

Nanowires possess a high aspect ratio, thus increasing the surface-to-volume ratio, leading to wonderful stabilities and superior gas-sensing properties. For instance, WO_3_ nanowires have been used for NO gas sensors, which showed high response (37) with fast response and recovery time (68 s/88 s) [77]. The enhanced gas-sensing performance was related to the large surface-to-volume ratio of the very fine nanowires. Compared with the increased surface-to-volume ratio, the improvement of gas-sensing performance caused by the size effect of semiconductor oxide is more significant [78]. This is due to the fact that the surface of a semiconductor oxide becomes more active as the size shrinks. In order to explore the dependence of the he gas-sensing performance on the nanowire diameter, Lupan et al. have prepared ZnO nanowires with different diameters (100–300 nm) for the detection of hydrogen [79]. The results indicated that ZnO nanowires with 100 nm in diameter showed the best response value to 100 ppm H_2_ at room temperature. The ZnO nanowires with smaller diameter meant that more atoms participated in the surface gas-sensing reactions, pointing out the importance of the size effect. Recently, arrays of 1D nanowires were found to benefit gas diffusion on account of their loose structural features. Qin et al. have prepared gas sensors based on aligned arrays of W_18_O_49_ nanowires [80]. Due to their beneficial sensing structure, the W_18_O_49_ nanowire arrays possessed wonderful NO_2_ sensing performance with high selectivity and fast response/recovery characteristics. The aligned nanowires arrays exhibited good interface performance with good adhesion and electrical contact, making a sensor with perfect stability. Besides, the intercrossing nanowire/nanowire junctions provided electrical paths for increasing the potential barrier height, contributing to outstanding changes in the resistance.

Nanotubes not only have good porosity but also a large surface area due to their hollow structures. Recently, nanotubes have been prepared by many methods, including template sol-gel processes, electrospinning, atomic layer deposition, and hydrothermal synthesis, etc. For instance, In_2_O_3_ nanotubes were fabricated by coaxial electrospinning in order to enhance their HCHO sensing properties. The nanotubes were composed of numerous In_2_O_3_ grains, which showed a rough surface [81]. It was found that the gas-sensing performance can be improved by adjusting the grain size. Moreover, the grain sizes of In_2_O_3_ nanotubes could be tuned by changing the calcination temperature. As the gas-sensing properties of HCHO gas were investigated, the gas sensor exhibited the highest response for In_2_O_3_ nanotubes calcined at 400 °C compared to In_2_O_3_ nanotubes calcined at 600 °C and 800 °C. The results indicated that gas-sensing performance increased with decreasing grain sizes at relatively low calcination temperature.

**Figure 7:**
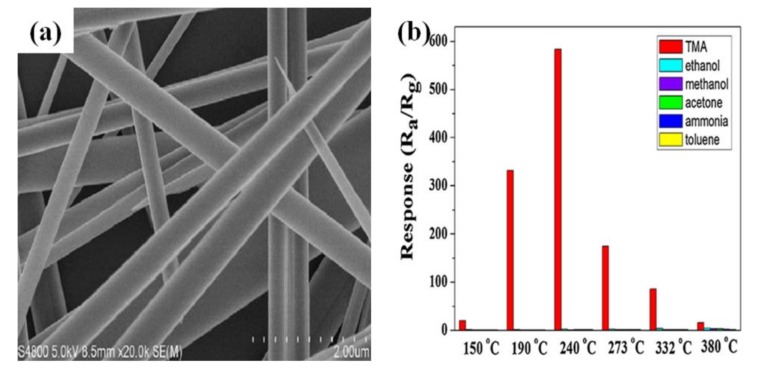
(**a**) High resolution FESEM image of ultra-long MoO_3_ nanobelts; (**b**) Response of the sensor based on ultra-long MoO_3_ nanobelts exposure of different gas at different temperatures. Reprint from [83] with permission. Copyright (2015) Elsevier.

Generally, ultra-long 1D nanostructured materials afford an opportunity for electron transport along the axial direction, contributing to enhanced gas-sensing characteristics of the semiconductor oxides. Apart from the special nanostructure, exposed specific crystal facets of semiconductor oxides can also lead to an improved gas-sensing properties [82]. For example, Yang et al. synthesized ultra-long single crystalline MoO_3_ nanobelts of about 200 µm in length and 200–400 nm in width, as shown in Figure 7a. The response increased with increasing operating temperature until the response reached a maximum value of 582 toward 50 ppm TMA at 240 °C (Figure 7b). The high response was ascribed to a direct path to electronic transmission along the axial direction provided by ultra-long MoO_3_ nanobelts, resulting in a rapid electron transfer [83]. In addition, due to the fact the (010) surface of MoO_3_ was exposed, it would be easier to form oxygen vacancies thus enhancing the activities for the improvement of the gas-sensing properties.

Among various 1D nanostructured materials for gas-sensing applications, researchers have been especially interested in nanofibers. Nanofibers can offer high specific surface area due to both a long length and small diameter. Optimizing the morphology of nanofibers is of importance for improving the performance of gas sensors. Khalil et al. have investigated how controlling the morphology could be an effective means to achieve the best gas-sensing performance of NiO nanofibers [84]. The morphologies of NiO nanofibers varied from rough and discontinuous to smooth and continuous with increasing NiAc/PVA ratios. As mentioned earlier, the sensor response increased with increasing surface area. At the same time, the gas-sensing performances of sensors are also affected by the inter-particle connectivity. Yoon et al. discussed the importance of inter-particle connectivity and showed that the response of nanofibers decrease with decreasing inter-particle connectivity [85]. Thus, NiO nanofibers obtained from 1.5 wt/wt precursors possessed the highest response to H_2_ and NH_3_, which was ascribed to a combination of high surface area and good inter-particle connectivity. The result suggested that the high surface area was as important as the inter-particle connectivity.

### 3.3. Two-Dimensional Sensing Materials

In recent years, 2D nanostructures have received strong research interest because of their unique morphologies. Thus, they can be used in many fields, such as gas sensors [86], lithium-ion batteries [87], supercapacitors [88], photoelectrochemistry [89], and photocatalysts [90]. Recently there have been a certain number of publications about 2D semiconductor oxides in gas sensors [91,92,93,94]. For example, small crystallite MoO_3_ nanoplates prepared by ultrasonic spray pyrolysis exhibited gas-accessible nanostructures with a relatively low detection limit (45 ppb TMA) [95]. The MoO_3_ nanoplate-based sensor showed ultrasensitivity and ultraselectivity to TMA. The ultrasensitivity to TMA was related to the thin nanosheets with large surface-to-volume ratio, inducing greater electron depletion and faster gas diffusion toward the entire surface of the MoO_3_ nanoplates. In addition, MoO_3_ as an acidic oxide had a high preference to react with basic gases, leading to ultraselectivity to TMA. In other words, the nature of TMA could lead to stronger chemisorption on the surface of MoO_3_, thereby increasing the electron transfer and improving the gas response.

Porous nanostructures will not only help gas interaction occurring on the surface but also facilitate the penetration of gas into the sensing material, relative to a dense structure. Porous nanostructures can be classified according to the pore size [96]. The size of micropore is smaller than 2 nm whereas macroporous nanostructures have pore widths larger than 50 nm. Mesoporous materials have pore diameters of 2–50 nm. In order to better improve the gas-sensing performance, mesoporous nanostructures and 2D nanosheets could be combined to exert their respective advantages. For example, Wang et al. have synthesized mesoporous In_2_O_3_ ultrathin nanosheets that presented superior response (213) as well as short response times (4 s) to NO_x_ at a relatively low operating temperature with a detection limit as low as 10 ppb [97]. The nanosheets with ultrathin thickness of 3.7 nm possessed a number of active sites, which lead to an increased response to NO_x_. Moreover, the mesoporous structures shorten the transport path and enhance gas diffusion. Thus, the advantaged combination achieves the improvement of the gas-sensing performance.

**Figure 8:**
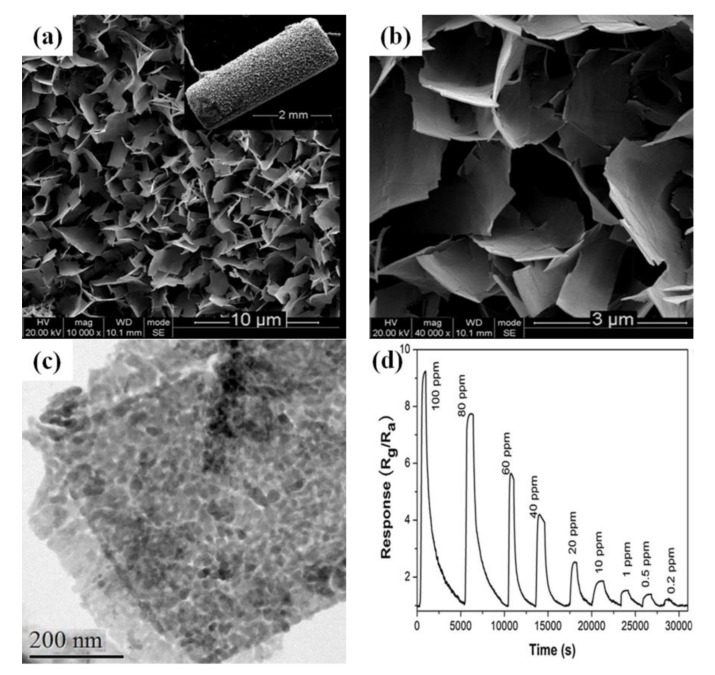
(**a**) SEM image of samples with magnification of 10,000 times (inset indicates the panoramic image of the Co_3_O_4_ on the surface of alumina tube); (**b**) SEM images with magnification of 40,000 times; (**c**) TEM image of Co_3_O_4_ nanosheet; (**d**) Dynamic response-recovery curve of the sensor based on Co_3_O_4_ nanosheet arrays to NH_3_ gas at the room temperature. Reprinted from [98] with permission. Copyright (2016) Elsevier.

In addition, network-like structures can contribute to rapid gas adsorption on the entire surface of a semiconductor oxide, leading to significantly increased gas-sensing performance. Networked nanosheet arrays of Co_3_O_4_ were prepared for the detection of NH_3_ [98]. The morphologies of Co_3_O_4_ crossed nanosheets arrays are shown in Figure 8a,b. The inset of Figure 3a showed the corresponding Co_3_O_4_ crossed nanosheets grown directly on substrates. The crossed nanosheets with cavities offered rapid electrical channels, ensuring that the network-like structure presented good gas-sensing performance. Besides, it could be seen that the Co_3_O_4_ nanosheet was composed of many small grains, as shown in Figure 8c. This indicated that the nanosheet surface structure facilitated gas diffusion. Figure 8d shows the response/recovery curves for different concentrations of NH_3_ gas. As can be seen, the Co_3_O_4_ nanosheet arrays possessed good responses even at a relatively low concentration of 0.2 ppm NH_3_ due to the fact the nanosheet intersect with each other with a superior open structure. The special structure not only prevents aggregation but also supplies a larger effective surface to react with target gases, leading to an enhanced gas response.

Semiconductor oxide-based thin films are also a kind of 2D sensing material, and the surface roughness of the films can enhance the sensing properties of gas sensors. Escalante et al. prepared ZnO films which displayed good gas-sensing properties toward 200 ppm CO at an operating temperature of 300 °C by single source chemical vapor deposition [99]. The plate-like structure on the surface of ZnO film displayed morphology of surface roughness, determining the effective surface area of the film and resulting in a significant impact on the gas response. In addition, the ZnO film contained plate-like structures with polycrystalline formation and thus gas molecules can be stored in the boundary and interface of grains, leading to an enhanced gas-sensing performance. Aside from these examples, the gas-sensing performances of 2D nanostructures with various morphologies were reported in many references (Table 2) and will be summarized in this review.

### 3.4. Three-Dimensional Sensing Materials

Three-dimensional hierarchical nanostructures are assembled from low dimensional nanomaterials, including 0D nanoparticles, 1D nanorods and 2D nanosheets. Three-dimensional hierarchical nanostructures are considered to be a more potential sensing material for detecting toxic gases than 0D, 1D, 2D nanostructures, because they can provide a large surface area, abundant active sites and fast interfacial transport. Hence, many semiconductor oxides with three-dimensional hierarchical nanostructures have been prepared to detect gases.

The conglomeration of 0D nanoparticles has been deemed as an obstacle in the improvement of gas-sensing performance, so researchers put forward an idea that 0D nanoparticles as building blocks be assembled into three-dimensional hierarchical nanostructures, thus improving the gas-sensing performance. For instance, Wu et al. synthesized a three-dimensional hierarchical Co_3_O_4_ nanostructure that was composed of 0D nanoparticles with an average size of 20 nm [109]. The unique 3D structure exhibited superior response to 100 ppm NH_3_ gas, rapid response time of 2 s and a detection limit as low as 0.5 ppm at room temperature. This was because of the fact that the hierarchical architecture provided more channels for electron transmission and facilitated gas diffusion toward the Co_3_O_4_ surface as well as the bulk. The connectivity between the particles in the 3D hierarchical Co_3_O_4_ materials offered higher density of defects, which ensured the fast adsorption into deeper regions of the sensing materials.

Generally, 1D nanorods are an important assembly element that can facilitate fast electron transfer and increase more the sensing activity sites due to their high aspect ratio. Very recently, a 3D SnO_2_ hierarchical nanostructure with nanorod assembled elements showed remarkably improved response compared to a commercial SnO_2_ nanopowder, which was ascribed to a dramatic enhancement of surface accessibility and gas diffusion resulting from the unique loose and porous 3D structure [110]. Further, Li et al. have prepared loose-, moderate- and close-assembled SnO_2_ nanorod-assembled urchins by tuning their assembly densities [111]. The results indicated that the close-assembled urchins take more time in gas diffusion due to their small pores and long diffusion lengths. Besides, the poor contact among nanorods in the loose-assembled urchins hinders efficient charge transfer. A comparative study indicated that moderate-assembled urchins exhibited the best gas-sensing performance toward ethanol, meaning that there might be an optimized assembly density range which offers enough space for gas diffusion as well as a sufficient contact network for charge transfer.

Two-dimensional nanosheets are also used as building blocks in hierarchical nanostructures, because they have large specific surface areas. The flower-like Co_3_O_4_ structures were composed of single-crystalline nanosheets, and each nanosheet contained numerous pores with an ultrahigh density [112]. The unique structure showed not only superior response and short response/recovery time, but also excellent stability toward xylene compared with commercial Co_3_O_4_. This may be attributed to single-crystalline nanosheets with good stability as well as a porous structure with a high permeability. Moreover, the flower-like structures provided much more contacts between gas molecules and semiconductor oxides and the enlarged specific surface area offered not only efficient diffusion paths, but also more active sites for gas absorption/desorption.

**Figure 9:**
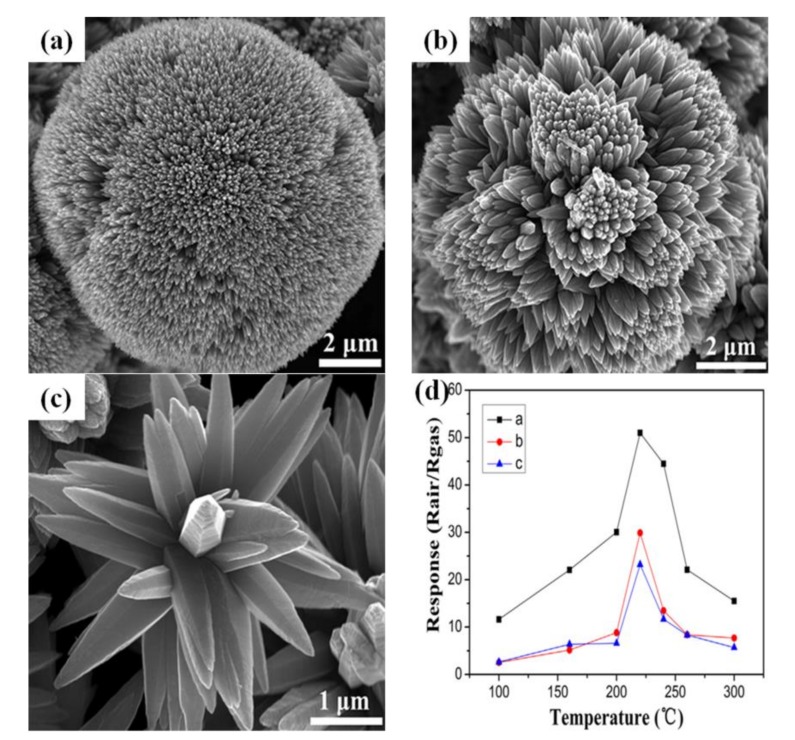
(**a**) FESEM image of sphere-like ZnO microstructure; (**b**) FESEM image of cauliflower-like ZnO microstructure; (**c**) FESEM image of sisal-like ZnO microstructure; (**d**) The response of different hierarchical ZnO microstructures sensors to 1000 ppb H_2_S at different operating temperature (100–300 °C). Reprinted from [113] with permission. Copyright (2015) Elsevier.

In order to gain a better understanding of the morphology-dependent gas-sensing performance of three-dimensional hierarchical nanostructures, Diao et al. have prepared three different hierarchical ZnO microstructures (sphere-like, cauliflower-like and sisal-like) to detect H_2_S, as shown in Figure 9a–c [113]. The response of a ZnO nanowire-assembled spherical structure was much higher than that of the cauliflower-like structure and sisal-like structure, respectively, for 1000 ppb of H_2_S as shown in Figure 9d. The higher response of sphere-like microstructures might be ascribed to the nature of the particle-to-particle contacts. Herein, particle-to-particle contacts result from nanowire-to-nanowire contacts, however, the effective particle-to-particle contacts had an obvious decrease owing to the low dimension nanowires, but the sphere-like microstructures were covered by very large amounts of nanowires, thus enhancing particle-to-particle contacts and motivating the changes of sensor resistance. Consequently, sphere-like microstructures presented the best sensing performance.

More recently, 3D macro-mesoporous structure was thought to be potential candidates for gas sensor applications due to their high porosity and less agglomerated configuration. The structure facilitates gas permeation and shortens gas diffusion length, thus contributing to a greatly improved gas-sensing performance. Liu et al. synthesized 3D interconnected macro-mesoporous ZnO nanostructures with different macropore sizes [114]. It was found that 3D ZnO nanostructures with interconnected channels between two mesoporous exhibited excellent sensing performances toward acetone, whereas ZnO nanoparticles showed a lower response due to a tortuous and long pathway. In addition, the largest macropore in the 3D ZnO nanostructures provided the largest cavities and wonderful channels for gas molecules, leading to the best response to 100 ppm acetone with a value of about 137. In order to prove the selectivity of 3D ZnO nanostructures, the sensing material was reacted with acetone and methanol, respectively.

The results revealed a prominent selectivity to acetone compared with methanol, owing to a higher electron density and more released electrons. To date, there are many articles published on the topic of fabricating 3D nanostructures with various morphologies. The gas-sensing performances of 3D nanostructures with various morphologies are summarized in Table 3.

## 4. Hollow Semiconductor Oxide Structures for Gas-Sensing Performance

**Figure 10:**
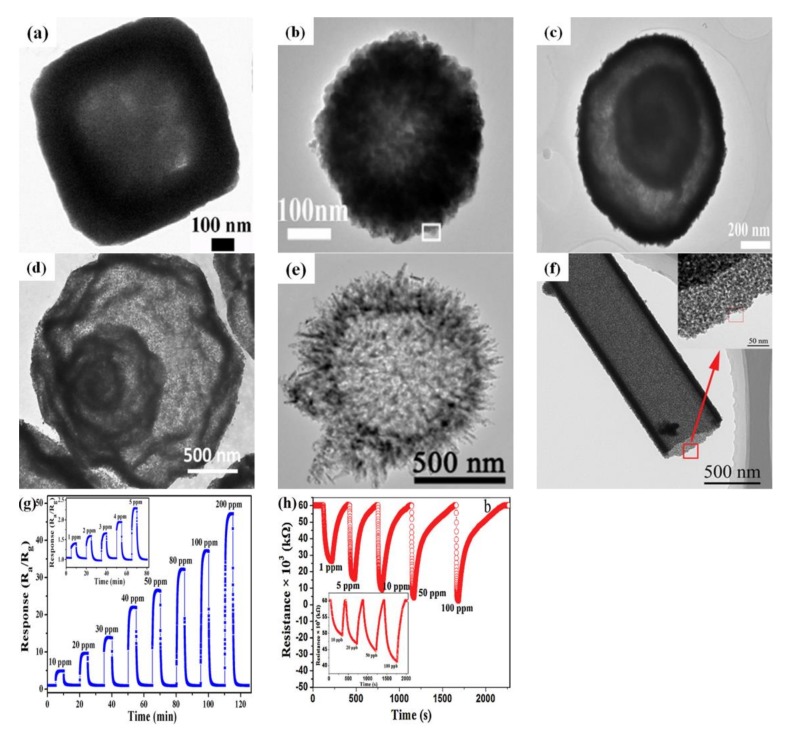
(**a**) TEM image of ZnSnO_3_ hollow cube; (**b**) TEM image of SnO_2_ hollow spheres; (**c**) TEM image of multi-shelled ZnO hollow sphere; (**d**) TEM image of multi-shelled Cr_2_O_3_ hollow sphere; (**e**) TEM image of ZnFe_2_O_4_ hollow microsphere; (**f**) TEM image of SnO_2_ hollow microtube; (**g**) Dynamic response transient of ZnFe_2_O_4_ hollow microsphere to different concentrations of acetone at 215 °C; (**h**) The relationship between the responses of SnO_2_ microtubes sensor and HCHO concentration at 92 °C. Reprinted from [133,134,135,136,137,138] with permission; (**a**) [133] Copyright (2016) Elsevier; (**b**) [134] Copyright (2016) American Chemical Society; (**c**,**d**) [135,136] Copyright (2017,2014) Elsevier; (**e**,**g**) [137] Copyright (2015) American Chemical Society; (**f**,**h**) [138] Copyright (2017) Elsevier.

A hollow structure may facilitate gas diffusion of a semiconductor oxide, which can effectively improve the performance of sensors. Compared with solid nanostructures, nanostructures with hollow interiors achieve better characteristics by introducing the inner-surface into the reactions. Therefore, hollow semiconductor oxide structures which can absorb gases into the inner-surface/outer-surface are widely used in the gas sensor field [128,129,130,131,132]. Figure 10a–f presented different morphologies and structural characteristics of some hollow nanostructures [133,134,135,136,137,138].

In addition, Figure 10g,h showed the sensing properties of ZnFe_2_O_4_ hollow microspheres and SnO_2_ hollow microtubes, respectively. It could be seen that the distinctive structure was fully adopted in the gas sensors, and the gas sensor performance was improved to obtain high responses and low detection limits. Recently, the gas-sensing performances hollow structures have been reported in a vast amount of publications, as shown in Table 4, but researchers are still eager to synthesize novel hollow structures to further improve the performance of gas sensors. Furthermore, the hollow nanostructures can offer an additional opportunity to adjust the characteristics by controlling the morphological parameters of crystal size, shell thickness and hole diameter.

The sensing performance of gas sensors can be improved though controlling the growth of a semiconductor oxide into a special morphology, so novel hollow structure morphologies can be introduced, such as flower-like, hollowed-out and microcubes. These particular architectures, which can provide good accessibility and more active sites for gas adsorption/desorption, have unique advantages in gas-sensing applications. Xu et al. have prepared a hollow flower-like porous In_2_O_3_ nanostructure by a solvothermal method [152]. The flower-like nanostructure was composed of nanosheets, and the nanosheets consisted of abundant nanoparticles. These nanoparticles with nanosize quantum confinement effects formed many active sites that facilitated gas adsorption on the surface. The hollow structure provided more passageways for accelerating gas accessibility and transmission on the inner and outer surface of the In_2_O_3_ nanostructure, so a unique morphology is conducive to improving the sensing performance of semiconductor oxides.

Moreover, the hollowed-out structure can endow semiconductor oxides with distinct porous natures, contributing to performance improvements of the gas sensor. Due to their open diffusion channels, hollowed-out structures can afford more convenient pathways to gas molecules. For instance, Tan et al. have reported that the hollow Co_3_O_4_ microspheres assembled from nanosheets exhibited high response with a value of 38.8 to 100 ppm ethanol due to the hollowed-out structure [153]. The hollowed-out structure could guarantee that large quantities of oxygen molecules and ethanol gases could permeate into the active sites on the entire surface and inner-surface. In addition, the hollowed-out Co_3_O_4_ microspheres presented ultrafast response and recovery time of 0.1 s and 0.7 s. This was a rare instance, in which response and recovery time were both less than 1 s. The ultrafast response/recovery speed was ascribed to the porous nanosheets and the nature of Co_3_O_4_. On the one hand, the porous nanosheets composed by interconnected nanoparticles have greater pore diameter than ethanol molecular clusters which endows them with higher permeability and faster electron transmission. On the other hand, Co_3_O_4_ has a high catalytic activity boosting reaction rate, therefore, the hollowed-out Co_3_O_4_ microspheres have an extraordinary gas-sensing performance.

Recently, researchers have taken an intense interest in the hollow microcube with well-defined interior voids. Compared to spheres with point-to-point contacting, microcubes with the face-to-face contacting increase contact area and improve performance of gas sensor. Zhang et al. reported that hollow MoO_3_ microcube exhibited obviously enhanced response to 100 ppm ethanol with the value of about 78 and response/recovery time of 15 s/5 s [154]. This was related to the advantages of large surface area resulting from the hollow microcubes. That is, more oxygen molecules absorbed on the surface of the microcubes in the air and reacted with ethanol, contributing to a high response value. Moreover, there was a wider conductive path when the captured free electrons were released to the MoO_3_ conduction band, which was ascribed to microcubes with face-to-face contact, so the MoO_3_ microcubes with a larger contact area displayed superior gas-sensing properties and faster reaction rates.

In addition, the gas response of hollow semiconductor oxides is also influenced by their morphological parameters. Thus, Li et al. have discussed the influence of the crystal size and the shell thickness on the gas-sensing performance of SnO_2_ hollow microspheres [155]. The obtained results indicated that the acetone sensing performance increased with the decrease of the crystal size and the shell thickness of the SnO_2_ hollow microspheres. The small crystal size and the thin shell thickness favored the adsorption and desorption of gas molecules on the surface, therefore, a controlled morphology can achieve an optimal gas-sensing performance.

However, these studies focus on the 3D nanostructure, but 1D nanostructures also can form a hollow interior space, and the hole diameter is one of factors influencing the gas-sensing performance of 1D hollow nanostructures. Compared with conventional solid fibers, hollow fibers have unique structural characteristics. In the hollow fibers, the hole diameter directly determines the area of the inner surface, thus changing the sensing performance. Accordingly, Katoch et al. discussed the influence of the hole diameter on the sensing performance of ZnO hollow fibers. The gas-sensing performance of hollow nanofibers can be improved by tuning the hole diameter. It was reported that the specific surface area increased with the decrease of the hole diameter, which was responsible for the performance of the gas sensors. In addition, ZnO hollow fibers with smaller hole diameter are much more conducive to obtaining high performances [156].

## 5. Core-Shell Structure of Semiconductor Oxide for Gas-Sensing Performance

**Figure 11:**
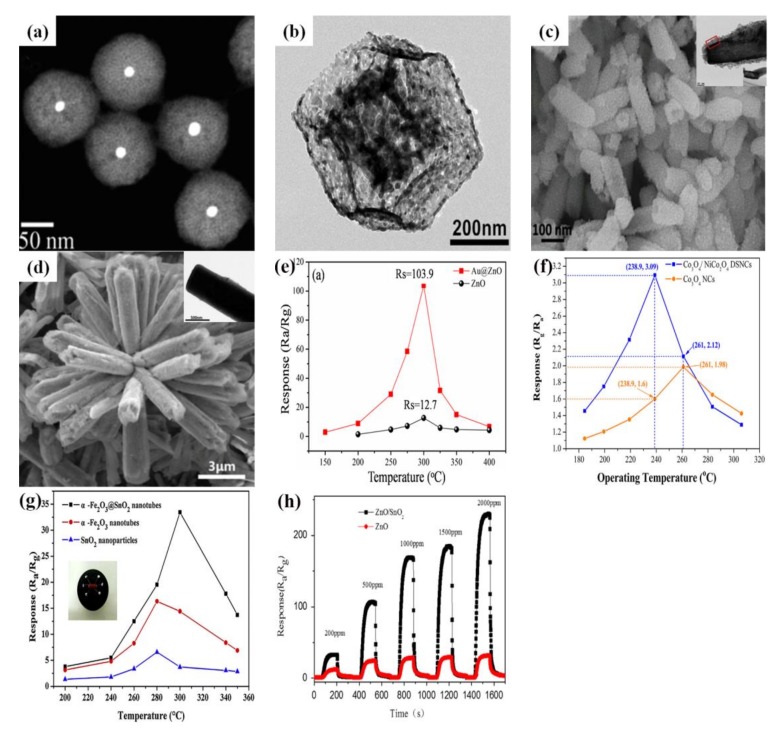
(**a**) TEM image of Au@ZnO core-shell nanoparticles; (**b**) TEM image of Co_3_O_4_/NiCo_2_O_4_ core-shell nanocages; (**c**) SEM and TEM images of α-Fe_2_O_3_@SnO_2_ core-shell nanotubes; (**d**) SEM and TEM images of ZnO/SnO_2_ core-shell nanorods; (**e**) Response of sensors based on the pure ZnO and Au/ZnO core-shell nanoparticles to 100 ppm of H_2_; (**f**) Response of sensors based on the Co_3_O_4_/NiCo_2_O_4_ core-shell nanocages and Co_3_O_4_ nanocages to 100 ppm acetone; (**g**) Variations of response (R_a_/R_g_) for the α-Fe_2_O_3_/SnO_2_ heterostructures, the pristine α-Fe_2_O_3_ nanotubes and the SnO_2_ nanoparticles to 100 ppm acetone at different operating temperatures; (**h**) the transient response curves of sensors (pure ZnO and ZnO/SnO_2_) with different concentration of ethanol at 300 °C. Reprinted from [158,159,160,161] with permission; (**a**,**e**) [158] Copyright (2015) American Chemical Society; (**b**,**f**) [159] Copyright (2017) Elsevier; (**c**,**g**) [160] Copyright (2015) Elsevier; (**d**,**h**) [161] Copyright (2015) Elsevier.

Core-shell semiconductor oxide structures, as a kind of very potential sensing material, have been gaining extensive attention for gas sensors. The unique nanostructure plays a key role in the performance of the gas sensor. The core-shell structures are divided into seven types, depending on the type of core and shell [157]. Core-shell structures have various gas-sensing performances because of the different configurations based on the core and the shell. In addition, core-shell nanostructures ensure a huge surface area and rapid gas diffusion, which is preferable to improve the gas-sensing characteristics of the sensor. Recently, many studies based on core-shell structure materials and their gas-sensing performances have been reported, as shown in Figure 11 [158,159,160,161]. As can be seen in some studies that contrast the responses, it is quite obvious that the gas-sensing performance is significantly improved with the introduction of core-shell structures. That is to say, the morphological characteristics influence the performance of gas sensors, so it is very necessary to summarize the sensing performances of core-shell structures, as shown in Table 5.

The yolk-shell nanostructure is considered to be a specific kind of core-shell structure with a unique core@void@shell configuration. It provides more space for gas sensor applications due to the particularity of the structure. Since then, more and more researchers have paid attention to the preparation of yolk-shell nanostructures in order to obtain superior gas-sensing performance. For instance, Wang et al. have prepared solid, hollow, yolk-shell α-Fe_2_O_3_ nanospheres by adjusting the reaction time [175]. The yolk-shell α-Fe_2_O_3_ nanospheres exhibited better gas-sensing performance to ethanol than the other two nanospheres due to the unique nanostructure. This was related to the inner shell having a hollow structure, leading to the adsorption of more gas molecules in the gas-sensing reaction. Moreover, the void between the outer shell and the inner core ensured that α-Fe_2_O_3_ nanospheres had a larger specific surface area. In the meantime, the porous outer shell could accelerate the permeability and diffusion of ethanol gases, thus enhancing the sensing performance.

More recently, Kim et al. have fabricated an enhanced gas sensor based on triple-shelled WO_3_ yolk-shell spheres [176]. In order to verify the excellent gas-sensing performance of multi-shelled WO_3_ yolk-shell spheres, dense WO_3_ spheres and double-shelled yolk-shell WO_3_ spheres were also prepared for the detection of NO_2_ gas. The sensor response towards 50 ppb NO_2_ at an operating temperature of 100 °C was increased from 8.9 for dense WO_3_ spheres and 57.3 for double-shelled yolk-shell WO_3_ spheres to 100 for triple-shelled WO_3_ yolk-shell spheres. After the comparison, it could be known that the response of yolk-shell spheres was significantly higher than that of dense spheres due to the increased gas accessibility. In addition, the thin and multi-shelled yolk-shell morphologies resulted in higher permeability compared with double-shelled yolk-shell spheres, contributing to the enhanced response to ppb level NO_2_. Thus, different types of structural characteristics may account for the response differences, revealing that a unique morphology and structure can be very helpful in enhancing the gas response. Since then, Bing et al. have prepared unique SnO_2_ yolk-shell cuboctahedra with accessible surface [177]. The nonspherical yolk-shell structures exhibited a high response value of 28.6 and a fast response speed of 1.8 s towards 20 ppm toluene at 250 °C due to the high surface area as well as their penetrable shells and cores. Besides, the face-to-face contacting possessed a broader conductive channel for free electrons, leading to a lower height of the potential barrier. As a result, a high response value and a rapid response speed could be obtained simultaneously.

In general, core-shell structures are formed by two materials, combining the advantages of each other. Li et al. have prepared Au/In_2_O_3_ core-shell microstructures, where the Au core was surrounded by a In_2_O_3_ shell layer with a thickness of about 50 nm [178]. According to the comparative gas response to HCHO gas between Au/In_2_O_3_ core-shell microstructures and pure In_2_O_3_ spheres, it was indicated that the Au/In_2_O_3_ composites showed enhanced sensing performance. The improved response was explained on the basis of Au nanoparticles with exceptional catalytic activities. What’s more, the loose and porous In_2_O_3_ shell could be boosted to increase the accessibilities of Au nanoparticles to HCHO gas molecules thus potentially triggering an increase in the response characteristics. More importantly, the formation of the metal-semiconductor junction induced a broader electron depletion layer and greater band bending compared with pure In_2_O_3_ spheres, so when exposed to HCHO gas, more captured electrons would be released back to the conduction band, leading to a sensing performance enhancement.

As we all know, high specific surface area signifies the production of more absorbed oxygen species in the gas-sensing reaction that subsequently interact with target gases. Qu et al. have prepared ZnO/ZnCo_2_O_4_ core-shell nanocages to detect xylene [179]. The ZnO/ZnCo_2_O_4_ core-shell nanocages exhibited remarkably enhanced gas-sensing performance in comparison with the single component semiconductor oxide, which was ascribed to the novel nanostructure with high specific surface area and the formation of a hole depletion layer caused by the Fermi level difference between ZnO and ZnCo_2_O_4_. In addition, the synergistic effect of ZnO and ZnCo_2_O_4_ as well as the catalytic effect of the cobalt oxides could improve the chemical activity of xylene gas, resulting in the increased response.

## 6. The Effect of Catalyst on Gas-Sensing Performance

Although the morphologies of semiconductor oxides have an effect on the gas-sensing properties, it is not enough. The improvement of the gas-sensing properties is also related to the use of additives in the semiconductor oxides. Thus, additives are often introduced into semiconductor oxides as sensitizers or promoters in order to improve gas-sensing performances due to their exceptional activities. It is noteworthy that the surface morphology of semiconductor oxide is also changed by adding a catalyst, thus forming a more advantageous surface for gas-sensing reactions. Consequently, the effective combination of morphology and catalysis will be beneficial to obtain a superior gas sensor with higher response, faster response speed, lower power consumption and lower limit of detection.

Commonly, a variety of strategies including element doping [180], noble metal loading [31] and surface functionalization [181] were used to obtain further enhanced gas-sensing performances. Among them, element doping is regarded as a facile and effective route to enhance gas-sensing performances. Han et al. have investigated the gas-sensing performances of Ce-doped In_2_O_3_ porous nanospheres toward 100 ppm methanol compared to pure In_2_O_3_ samples [182]. It was found that Ce-doped In_2_O_3_ porous nanospheres exhibited enhanced sensing performance due to the incorporation of the elemental Ce. The doping with Ce could greatly reduce the crystallite size in order to increase the specific surface area, forming an advantageous morphology that may help increase the electron transport. In addition, Ce ions released electrons back into the conduction band and the electronic properties are influenced by increasing the concentration of free electrons. Accordingly, Ce-doped In_2_O_3_ porous nanospheres are observed to give better gas-sensing performance than undoped In_2_O_3_ samples. Semiconductor oxides as sensitizers are also incorporated into semiconducting oxides, which induce more sensor selectivity to the target gas and reduce the power consumption. For example, Nasrabadi et al. have prepared Gd_2_O_3_-doped SnO_2_ nanoparticles that showed a maximum response at 150 °C, whereas pure SnO_2_ nanoparticles had the highest response at 250 °C [183]. Moreover, Gd_2_O_3_-doped SnO_2_ nanoparticles also exhibited ultrahigh selectivity to ethanol and the response toward ethanol was 355- and 1641-fold greater than the responses toward CH_4_ and CO, respectively. The catalytic oxidation properties arise from adding Gd_2_O_3_, which endowed Gd_2_O_3_-doped SnO_2_ nanoparticles surface with higher activities, so a higher amount of oxygen was adsorbed on the surface, inducing an improved response to ethanol.

Loading a noble metal catalyst (Pt, Pd, Au, Ag) is also a very effective way of improving gas-sensing performance [184,185]. Wang et al. have prepared Pt-loaded mesoporous WO_3_ and mesoporous WO_3_ for comparison [186]. The sensor response was increased from 6.72 for pure mesoporous WO_3_ to 13.61 for Pt-loaded mesoporous WO_3_. Thus, the sensor made of Pt-loaded mesoporous WO_3_ showed a strengthened gas-sensing performance toward ammonia compared to those made from pure mesoporous WO_3_, owing to the matrix and the catalyst. The matrix provided a mesoporous structure for promoting gas transport and diffusion, while, the catalytic properties of Pt additives accelerated the adsorption/desorption reactions of oxygen and ammonia gas. This indicated that the loaded noble metal had a significant impact on the gas-sensing performance. In general, noble metal loaded means mono-noble metal loaded, and the effect of multi-metallic loading has rarely been reported. Recently Fan et al. [187] prepared Pt/Au bimetallic nanoparticles loaded on ZnO nanorods, which exhibited better response to H_2_ than Pt-loaded ZnO and Au-loaded ZnO, owing to the effect of the bimetallic nanoparticles containing two mono-metal analogues. The bimetallic nanoparticles showed enhanced catalytic performances resulting from the synergistic effect of the two mono-metal nanoparticles, leading to improved adsorption properties and superior sensing properties.

**Figure 12:**
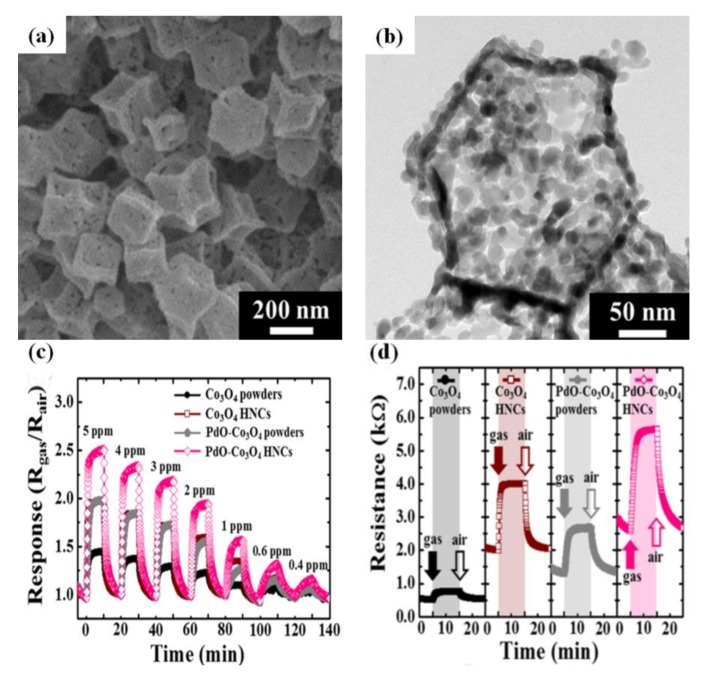
(**a**) SEM images of PdO-Co_3_O_4_ hollow nanocages; (**b**) TEM images of PdO/Co_3_O_4_ nanocages; (**c**) Dynamic acetone sensing transient properties of Co_3_O_4_ powders, Co_3_O_4_ hollow nanocages, PdO/Co_3_O_4_ powders, and PdO/Co_3_O_4_ hollow nanocages in the concentration range of 0.4–5.0 ppm; (**d**) Dynamic resistance transition toward 5 ppm of acetone molecules. Reprinted from [188] with permission. Copyright (2017) American Chemical Society.

In addition, it is worth mentioning that the gas-sensing performance can be substantially enhanced by surface functionalization. To investigate the effect of surface functionalization, Koo et al. have prepared PdO nanoparticles functionalized Co_3_O_4_ hollow nanocages by utilizing MOF as a template, as shown in Figure 12a,b. PdO nanoparticle-functionalized Co_3_O_4_ hollow nanocages displayed significantly enhanced acetone sensing performance owing to the effects of the hollow and porous structure as well as the functionalization by the nanoscale catalyst, which was superior to PdO/Co_3_O_4_ powders, Co_3_O_4_ hollow nanocages and Co_3_O_4_ powders, as shown in Figure 12c,d. The increase in the surface area could lead to the increment of reaction sites due to the unique structure. Moreover, PdO nanoparticles reacted with acetone molecules and released additional electrons to Co_3_O_4_, resulting in an increase in the resistance of the sensor [188].

## 7. Conclusions and Perspectives

With the continuing deterioration of the environment and peoples’ increasing awareness of the importance of protecting the environment, researchers are constantly looking for high performance gas sensors to detect deleterious gases. The morphologies of semiconductor oxides affect the properties of gas sensors, therefore, it is necessary to carry out an overview of different morphologies and their gas-sensing performances. This review gives a classification of morphologies, whereby semiconductor oxides can be defined as zero-dimensional, one-dimensional, two-dimensional and three-dimensional. The results indicate that semiconductor oxides with different morphologies each have their own unique advantages, contributing to the improvement of gas-sensing properties. Moreover, hollow structure and core-shell structure materials exhibit high gas-sensing performances due to their large specific surface areas, which allow the inner and outer surface to absorb more target gases.

Although sensors have been developed in recent years, the sensors with high performances still needs further develop by controlling the morphology and the nanostructure of semiconductor oxides. In the field of semiconducting oxide gas sensors, it can be predicted that potential strategies for enhancing the gas-sensing performance should involve several aspects: (1) novel semiconductor oxide nanostructures which may achieve ultrasensitive and ultraselective detection of gases; (2) hollow hierarchical of semiconductor oxides which provide a higher specific surface area to obtain more active sites for gas diffusion; (3) core-shell semiconductor oxide nanostructures that possess heterostructures, which can be regarded as a novel form of potential sensing materials. All this together suggests that the improvement of the performance of gas sensors will undoubtedly continue to be of great importance in the future.

## Figures and Tables

**Table 1:** Summary of the gas-sensing performances of 1D nanostructures with various morphologies to different gases.

Morphology	Gases	Materials	Conc. (ppm)	LOD (ppm)	Temp. (°C)	τ_res_ (s)	τ_rec_ (s)	Resp.	Ref.
nanowire	H_2_	CuO	100	NA	300	60	2	340 ^b^	[68]
nanowire	NO_2_	SnO_2_	2	NA	150	25 min	45 min	6.9 ^b^	[69]
nanowire	H_2_S	SnO_2_	10	NA	250	2.3	5 min	380 ^a^	[70]
nanorod array	O_2_	TiO_2_	4%	NA	RT	40	75	1.68 ^b^	[71]
nanorod	NO_2_	SnO_2_	5	0.03	150	5.9 min	2.6 min	5310 ^b^	[72]
nanorod	CH_4_	TiO_2_	60	NA	RT	45	33	6028 ^a^	[73]
nanofiber	ethanol	WO_3_	100	NA	350	NA	NA	62 ^a^	[74]
nanotube	methanol	ZnO	700	NA	NA	2.24 min	1.03 min	51.23% ^c^	[75]
nanobelt	NO	ZnO	50	0.5	RT	NA	NA	6.5 ^b^	[76]

^a^ S = R_a_/R_g_; ^b^ S = R_g_/R_a_; ^c^ (ΔR/R_a_) × 100%; LOD: limit of detection; NA = not available.

**Table 2:** Summary of the gas-sensing performances of 2D nanostructures with various morphologies to different gases.

Morphology	Gases	Materials	Conc. (ppm)	LOD (ppm)	Temp. (°C)	τ_res_ (s)	τ_rec_ (s)	Resp.	Ref.
nanosheet	acetone	Co_3_O_4_	100	1.8	150	NA	NA	11.4 ^b^	[100]
nanosheet arrays	NO_2_	ZnO	10	NA	180	3	12	20 ^b^	[101]
nanoplate	H_2_S	TiO_2_	100	NA	300	NA	NA	5.5 ^a^	[102]
nanoplate	NO_2_	WO_3_	100	1	100	NA	NA	131.75 ^b^	[103]
Nanoplate arrays	xylene	α-MoO_3_	100	NA	370	1	15	19.2 ^a^	[104]
thin film	NO_2_	WO_3_	1	NA	300	7	8	4.1 ^b^	[105]
thin film	acetone	ZnO	100	NA	280	6	18	30 ^a^	[106]
thin film	2-proponal	V_2_O_5_	5	NA	NA	3	10	176 ^a^	[107]
nanowall	NO_2_	ZnO	50	NA	450	23	11	6.4 ^b^	[108]

^a^ S = R_a_/R_g_; ^b^ S = R_g_/R_a_; LOD: limit of detection; NA = not available.

**Table 3:** Summary of the gas-sensing performances of 3D nanostructures with various morphologies to different gases.

Morphology	Gases	Materials	Conc. (ppm)	LOD (ppm)	Temp. (°C)	τ_res_ (s)	τ_rec_ (s)	Resp.	Ref.
microsphere	NO_2_	In_2_O_3_	1	NA	145	60	30	132 ^b^	[115]
microsphere	HCHO	SnO_2_	100	NA	200	17	25	38.3 ^a^	[116]
flower-like	TEA	α-MoO_3_	100	0.5	250	3	1283	416 ^a^	[117]
flower-like	ethanol	SnO_2_	500	NA	300	8	7	208 ^a^	[118]
flower-like	ethanol	NiO	400	NA	300	4	8	32 ^b^	[119]
flower-like	ethanol	ZnO	100	0.2	260	NA	NA	123 ^a^	[120]
flower-like	acetic acid	SnO_2_	100	NA	260	18	11	47.7 ^a^	[121]
flower-like	NO_2_	WO_3_	0.4	0.04	120	NA	NA	103 ^b^	[122]
rose-like	NO_2_	Cu_2_O	200	NA	340	NA	NA	6.8 ^b^	[123]
leaf-like	acetone	α-Fe_2_O_3_	200	NA	260	8	9	95.4 ^a^	[124]
urchin-like	ethanol	WO_3_	100	NA	350	28	12	68.56 ^a^	[125]
cactus-like	acetone	NiO	100	NA	260	24	39	13.51 ^b^	[126]
spindle-like polyhedra	HCHO	In_2_O_3_	20	NA	240	1	2	8.2 ^a^	[127]

^a^ S = R_a_/R_g_; ^b^ S = R_g_/R_a_; LOD: limit of detection; NA = not available.

**Table 4:** Summary of the gas-sensing performances of hollow structures to different gases.

Morphology	Gases	Materials	Conc. (ppm)	LOD (ppm)	Temp. (°C)	τ_res_ (s)	τ_rec_ (s)	Resp.	Ref.
hollow sphere	acetone	TiO_2_	100	NA	320	NA	NA	6.9 ^a^	[139]
hollow sphere	methanol	α-Fe_2_O_3_	10	1	280	8	9	25 ^a^	[140]
hollow sphere	NO_2_	WO_3_	0.1	NA	140	90	400	18 ^b^	[141]
hollow sphere	NO_2_	WO_3_	1	0.04	100	237	88	89 ^b^	[142]
hollow sphere	HCHO	SnO_2_	200	NA	300	NA	NA	9 ^a^	[143]
hollow sphere	ethanol	ZnSnO_3_	5	NA	280	NA	NA	4.5 ^a^	[144]
hollow sphere	ethanol	SnO_2_	200	NA	260	10	8	274.5 ^a^	[145]
multi-shelled hollow sphere	acetone	SnO_2_	200	NA	200	10	12	153 ^a^	[146]
hollow nanofiber	n-propanol	CuO	100	1	200	19.18	63	4.66 ^b^	[147]
hollow nanosheet	acetone	SnO_2_	50	NA	300	0.9	5.8	18.3 ^a^	[148]
hollow cauliflower-like	CO	WO_3_	300	NA	270	NA	NA	41.9 ^a^	[149]
hollow six-sided pyramids	ethanol	ZnO	200	NA	240	11	9	187 ^a^	[150]
hollow polyhedrons	acetone	ZnSnO_3_	50	NA	240	17	10	12.48 ^a^	[151]

^a^ S = R_a_/R_g_; ^b^ S = R_g_/R_a_; LOD: limit of detection; NA = not available.

**Table 5:** Summary of the sensing performances of core-shell structures to different gases.

Morphology	Gases	Materials	Conc. (ppm)	LOD (ppm)	Temp. (°C)	τ_res_ (s)	τ_rec_ (s)	Resp.	Ref.
core-shell nanoparticle	CO	Au/SnO_2_	200	NA	200	NA	NA	1.3 ^a^	[162]
core-shell nanoparticle	O_3_	Au/TiO_2_	2.5	0.36	RT	5	24	3.27 ^a^	[163]
core-shell nanofiber	TMA	In_2_O_3_/SnO_2_	10	NA	280	3	32	7.11 ^a^	[164]
core-shell nanofiber	HCHO	NiO/α-Fe_2_O_3_	50	1	240	2	9	12.8 ^a^	[165]
core-shell nanofiber	ethanol	SiO_2_/SnO_2_	200	NA	NA	13	16	37 ^a^	[166]
core-shell nanofiber	ethanol	ZnO/SnO_2_	100	NA	200	75	12	392.29 ^a^	[167]
core-shell nanowire	NO_2_	W_18_O_49_/TiO_2_	5	NA	RT	NA	NA	36.5 ^a^	[168]
core-shell nanosphere	H_2_S	CuO/NiO	100	NA	260	18	29	47.6 ^b^	[169]
core-shell nanosphere	NH_3_	Co_3_O_4_/SnO_2_	50	NA	200	4	17	13.6 ^a^	[170]
yolk-shell nanoparticle	ethanol	Pd/In_2_O_3_	5	NA	350	NA	NA	159.02 ^a^	[171]
yolk-shell nanoparticle	acetone	Au/ZnO	10	0.05	280	15	12	43 ^a^	[172]
yolk-shellnanostructure	H_2_S	MoO_3_/Fe_2_(MoO_4_)_3_	1	NA	70	20	70	1.7 ^a^	[173]
yolk-shellnanosphere	acetone	ZnFe_2_O_4_	50	0.5	200	NA	NA	28.5 ^a^	[174]

^a^ S = R_a_/R_g_; ^b^ S = R_g_/R_a_; LOD: limit of detection; NA = not available.
